# WGCNA analysis reveals hub genes in the *Hemarthria compressa* roots in response to waterlogging stress

**DOI:** 10.1038/s41598-025-94873-7

**Published:** 2025-04-22

**Authors:** Wenwen Li, Xiaoli Zhou, Minghao Qu, Yuqian Zheng, Bingna Shen, Bing Zeng, Yanlong Feng, Kaiyue Pang, Jiahai Wu, Bing Zeng

**Affiliations:** 1 College of Animal Science and Technology, Southwest Un Iversity, Chongqing, China; 2 Institute of Grass-Fed Livestock, Chongqing Academy of Animal Sciences, Chongqing, China; 3 Institute of Animal Husbandry and Veterinary Medicine, Guizhou Academy of Agricultural Sciences, Guizhou, China; 4 Chongqing University Herbivore Engineering Research Center, Chongqing, China

**Keywords:** *Hemarthria compressa*, Waterlogging stress, Transcriptome, Oxidative stress, Starch and sucrose metabolism, Root, Flooding, Plant breeding

## Abstract

*Hemarthria compressa* is a high-quality forage resource in China. In recent years, waterlogging has frequently occurred, adversely affecting the growth and development of *H. compressa*. In order to investigate the physiological and molecular response mechanisms of *H. compressa* under waterlogging stress and identify hub genes involved in waterlogging tolerance, *H. compressa* roots from the GY (waterlogging-tolerant) and N1291 (waterlogging-sensitive) cultivars were selected as experimental materials in this study. The physiological indexes of *H. compressa* were measured, and transcriptome sequencing was carried out after 8 h and 24 h of waterlogging stress, with 0 h used as the control group. Superoxide dismutase (SOD) and peroxidase (POD) activities were significantly increased in both GY and N1291 under waterlogging stress (*P* < 0.05). Weighted gene co-expression network analysis (WGCNA) identified a total of four modules significantly associated with waterlogging stress (*r*>|0.9|, *P* < 0.05). Gene ontology (GO) and Kyoto encyclopedia of genes and genomes (KEGG) enrichment results showed that differentially expressed genes (DEGs) were mainly enriched in the Starch and sucrose metabolism, Plant hormone signal transduction, Ribosome and Glutathione metabolism pathways. Seven hub genes were also retrieved, including *Cluster-38255.67514* and *Cluster-38255.80127*, potentially associated with waterlogging tolerance. It is related to the Ribosome pathway and participates in the process of anti-waterlogging regulation. The results of this experiment provide new insights into the response mechanisms of *H. compressa* to waterlogging stress and a theoretical framework for the effective selection and breeding of waterlogging-tolerant cultivars.

**Supplementary Information:**

The online version contains supplementary material available at 10.1038/s41598-025-94873-7.

## Introduction

In recent years, due to climate change, there has been an increase in global extreme rainfall intensity and frequent floods^1^. In 2023 alone, there will be 31 heavy rainfalls in Chongqing, China^2^. Floods have led to the inundation of land in many countries such as China, Australia, India, and Bangladesh, causing catastrophic consequences for crop production and food security, and leading to huge economic losses^3,4^. According to statistics, 12% of the world’s arable land isaffected by waterlogging, and flood events are expected to increase further in the coming decades^5^. Therefore, it is necessary to develop and breed waterlogging-tolerant crop cultivars to reduce agricultural economic losses and increase global crop yield.

O_2_ diffuses 10^4 ^times slower in water than in air, waterlogging stress can cause hypoxia or anoxic environment^6^. Plants use different morphological and physiological changes and transcriptional and metabolic regulation strategies to cope with hypoxic environment^7^. As the first organs to be persecuted by hypoxia, roots have crucial functions in plant waterlogging tolerance^8,9^. Studies have shown that some waterlogging-tolerant plants can alleviate stress after waterlogging through morphological adaptations such as the formation of adventitious roots (ARs) and aerenchyma^10–12^. *Melilotus siculus*, for example, is a waterlogging-tolerant legume that resists waterlogging stress by forming ARs and secondary aerenchyma^13^. Waterlogging induced *Polygonum hydropiper*to generate adventitious roots rapidly and promoted oxygen diffusion^14^. It has been proved that the process of plants receiving waterlogging signals to induce the formation of ARs and aerenchyma is related to ethylene (ET) accumulation^15,16^. In addition, plant anaerobic metabolism is activated under waterlogging stress to ensure survival in hypoxia / anoxia^17^. Peng et al.^18^ conducted waterlogging stress on the roots of two grape cultivars with different waterlogging tolerance and found that the regulatory mechanism from aerobic to anaerobic fermentation was partially absent in waterlogging-sensitive cultivars under anoxic conditions. Although anaerobic metabolism can briefly maintain energy supply and alleviate energy deficit, it consumes large amounts of carbohydrates^19^. With the prolongation of waterlogging, ethanol, reactive oxygen species (ROS), and other toxic compounds accumulate in plants, leading to cell apoptosis and even plant death^20,21^. ROS scavenging system usually includes an antioxidant enzyme system (such as Superoxide dismutase(SOD), Peroxidase (POD), Catalase (CAT), Ascorbate peroxidase(APX)) and endogenous non-enzymatic antioxidant system (such as Glutathione (GSH))^22^. Salah et al.^23^ used exogenous γ-aminobutyric acid to treat *Zea mays* and found that γ-aminobutyric acid could enhance the waterlogging tolerance of *Zea mays* by increasing the activities of antioxidant enzymes such as SOD, POD, CAT and APX. Liu et al.^24^ found that the activities of POD and APX in *Kandelia obovata* increased significantly under waterlogging stress, which helped to maintain the dynamic balance of ROS content in vivo.

In recent years, transcriptomics has been widely used to study the molecular mechanism of plant response to waterlogging stress^25,26^. WGCNA identifies biologically significant co-expression modules and core genes by associating them with target traits/phenotypes^27^. In recent years, WGCNA has been used to identify and mine co-expression modules and hub genes involved in the responses and adaptation to abiotic stresses in crops such as *Oryza sativa*^12^, *Triticum aestivum*^28^, and *Zea mays*^29^. Chen et al.^30^ used WGCNA to screen for hub genes associated with the genotype, stress duration, and leaf age in two *Hordeum vulgare* cultivars differing in waterlogging tolerance. Li et al.^31^ revealed the candidate genes *SUS* and *PDC* related to waterlogging response in the waterlogging-tolerant plant *Actinidia valvata* through WGCNA, and found that these genes were related to sucrose metabolism and fermentation.

*H. compressa* is a perennial high-quality forage grass of the *Poaceae *family, with a high yield, fast growth rate, strong resistance, and regeneration ability. It is mainly distributed in southwest China and is one of the most important and widely planted and utilized forage grasses in this region^32^. Forage grass will encounter various environmental conditions that hinder growth and survival in its life cycle. Waterlogging is one of the most common abiotic stresses in the growth and development of *H. compressa*. The increase of waterlogging intensity seriously affected the growth and development of *H. compressa *and reduced the quality and yield^33,34^. Currently, most studies on *H. compressa*adaptation and tolerance to abiotic stresses have focused on heavy metal stress, drought stress, and so on^35–37^. Studies on *H. compressa *genomics have mainly focused on simple sequence repeat (SSR) and single nucleotide polymorphism (SNP) molecular markers^38,39^. Little has been reported on the transcriptome and co-expression network analysis of *H. compressa* under waterlogging stress. Although the above studies provide a certain reference and direction for revealing the response mechanism of *H. compressa* to waterlogging stress, the abiotic stress response mechanism of plants is an intricate biological process, and there are certain differences in the waterlogging stress response mechanism among plant species with distinct growth habits. In our previous study, we used phenotypic and physiological characteristics to compare and identify waterlogging-tolerant “Guang yi” (GY) and waterlogging-sensitive N1291, but did not clarify the molecular mechanism and excavate the core genes of waterlogging tolerance.

Given this, this study selected two cultivars of *H. compressa* with different waterlogging tolerance, GY and N1291, to sequence and analyze the transcriptome of *H. compressa* roots at different waterlogging stages. WGCNA analysis was used to predict the molecular mechanism of *H. compressa* in response to waterlogging stress, the waterlogging tolerances hub genes were excavated, and the pathways and hub genes that help *H. compressa* adapt to and enhance waterlogging tolerance were actively elucidated. The results of this study can provide new genetic resources for *H. compressa* breeding in the future, and improve the adaptability and yield of *H. compressa* under floods.

## Results

### Physiological changes in *H. compressa* roots under waterlogging stress

**Fig. 1:**
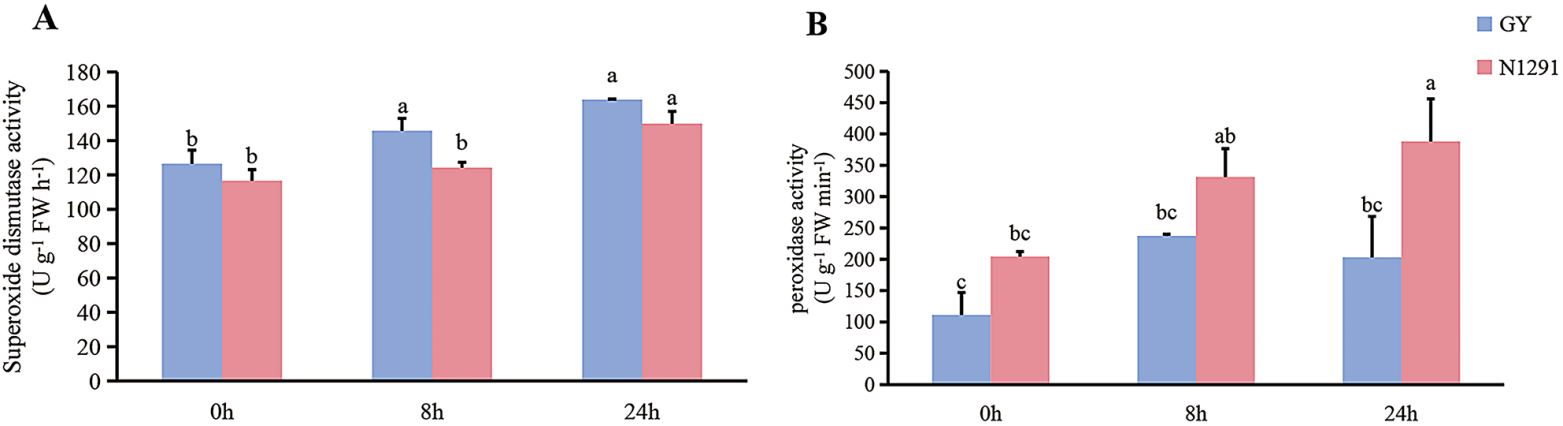
Changes in physiological indices of the *H. compressa* roots after 0 h, 8 h and 24 h of waterlogging stress. (**A**) Superoxide dismutase activity. (**B**) peroxidase activity. Different lowercase letters (a-c) indicate significant differences (LSD, *P* < 0.05).

The SOD activity in the roots of GY and N1291 showed a gradual increase with the increase of waterlogging stress period, and the SOD activity of GY was much greater than that of N1291 during the entire waterlogging stress period. Compared to 0 h, the SOD activity of GY increased significantly after 8 h and 24 h of waterlogging (*P* < 0.05). In contrast, no significant differences in SOD activity were observed in N1291 after 8 h of waterlogging compared to 0 h. However, there was a significant increase (*P* < 0.05) in SOD activity after 24 h of waterlogging (Fig. 1A). As the waterlogging stress time increased, the POD activity in the roots of GY showed an initially increasing and then decreasing trend. Specifically, compared to the control group, the POD activity of GY increased significantly after 8 h of waterlogging treatment (*P* < 0.05). In contrast, with the increase of waterlogging stress time, the POD activity of GY roots increased first and then decreased. The POD activity of GY was not significantly different from that of the control group after 8 h and 24 h of waterlogging treatment. The POD activity of N1291 increased gradually, and the POD activity of N1291 was significantly higher than that of the control group after 24 h of waterlogging stress (*P* < 0.05) (Fig. 1B).

### Transcriptome sequencing and assembly

In order to better understand and determine the key genes of *H.compressa* in response to waterlogging stress, high-throughput RNA sequencing (RNA-Seq) was performed on the roots of GY and N1291 (0 h, 8 h, 24 h) using the Illumina platform. A total of 403,903,531 paired end (PE) original reads of 150 bp were generated from 18 samples (6 groups × 3 replicates). After filtering low-quality and N-containing reads, 393,344,027 high-quality clean reads were obtained. The error rate was below 0.03%, the average Q20 and Q30 values were 96.93% and 92.08%, respectively, and the average GC content was 54.57% (Fig. S1). The assembly of clean reads produced 256,550 unigenes. The assembly length of unigenes was concentrated in the range of 300–1000 bp, with an average length of 862 bp and an N50 length of 1138 bp (Table S1). Therefore, the transcriptome sequencing results were of high quality and were utilized for subsequent analysis.

### Analysis of differentially expressed genes

**Fig. 2:**
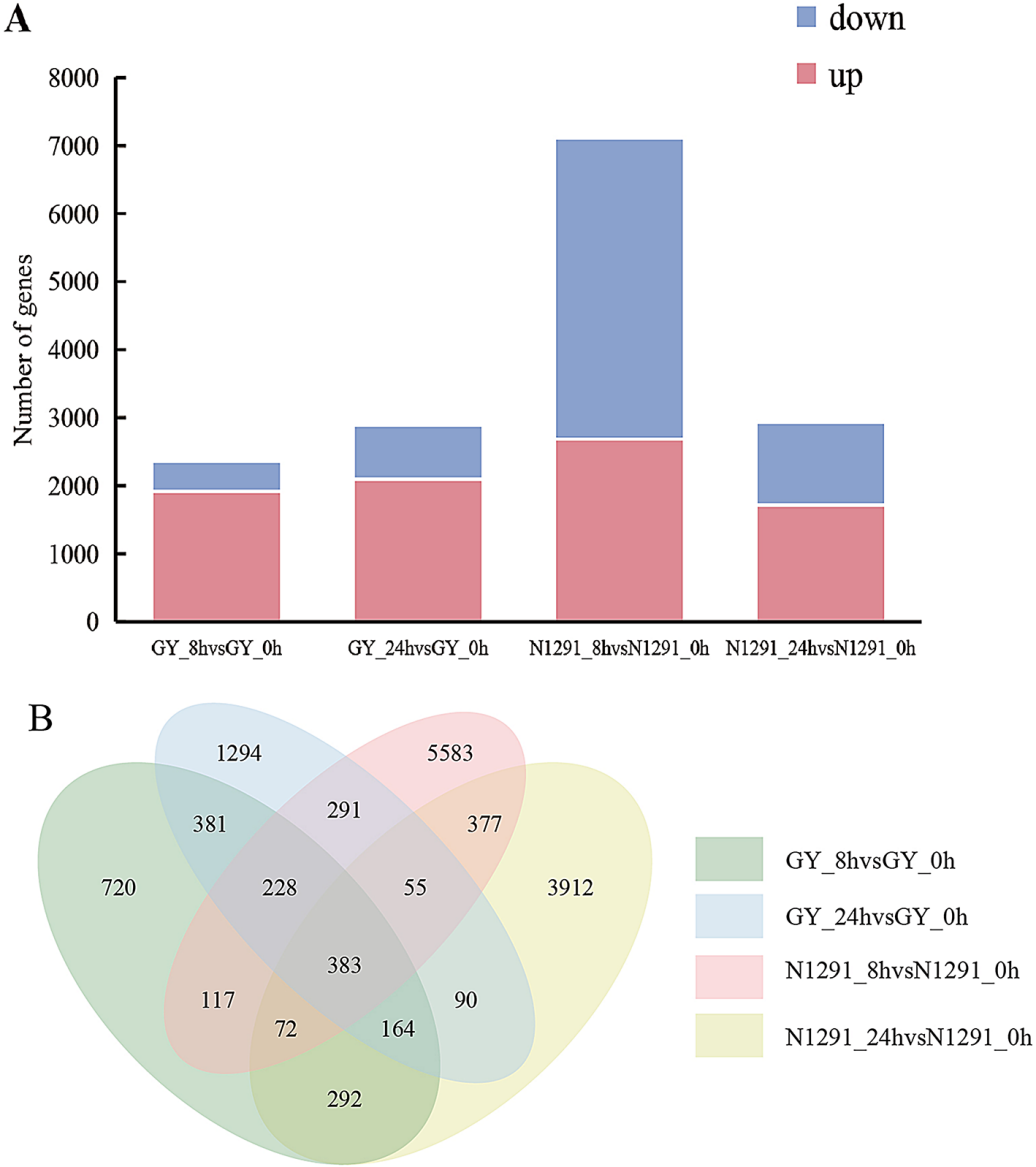
Analysis of differentially expressed genes. (**A**) Statistics on the number of differentially expressed genes that were up- and down-regulated. (**B**) Venn diagram of differentially expressed genes in different treatments. The non-intersection part represents the number of genes unique to the comparison group, and the intersection part represents the number of genes shared by the comparison group.

In this study, DEGs were screened with a threshold of |log_2_ (fold-change) |>1.5 and padj < 0.05. Compared with 0 h, a total of 2358 DEGs (1917 up-regulated and 441 down-regulated) were identified after 8 h of waterlogging stress in GY, and a total of 2886 DEGs (2097 up-regulated and 789 down-regulated) were identified after 24 h of waterlogging stress. In N1291, a total of 7106 DEGs (2685 up-regulated and 4421 down-regulated) were identified after 8 h of waterlogging stress, while a total of 2937 DEGs (1717 up-regulated and 1220 down-regulated) were identified after 24 h of waterlogging stress (Fig. 2A). According to the results of the Venn diagram, there were 383 common DEGs in GY and N1291 under waterlogging stress. Among them, 1156 and 2050 DEG were commonly expressed at both 8 h and 24 h of waterlogging stress, in GY and N1291, respectively (Fig. 2B).

### Weighted gene co-expression network analysis

**Fig. 3:**
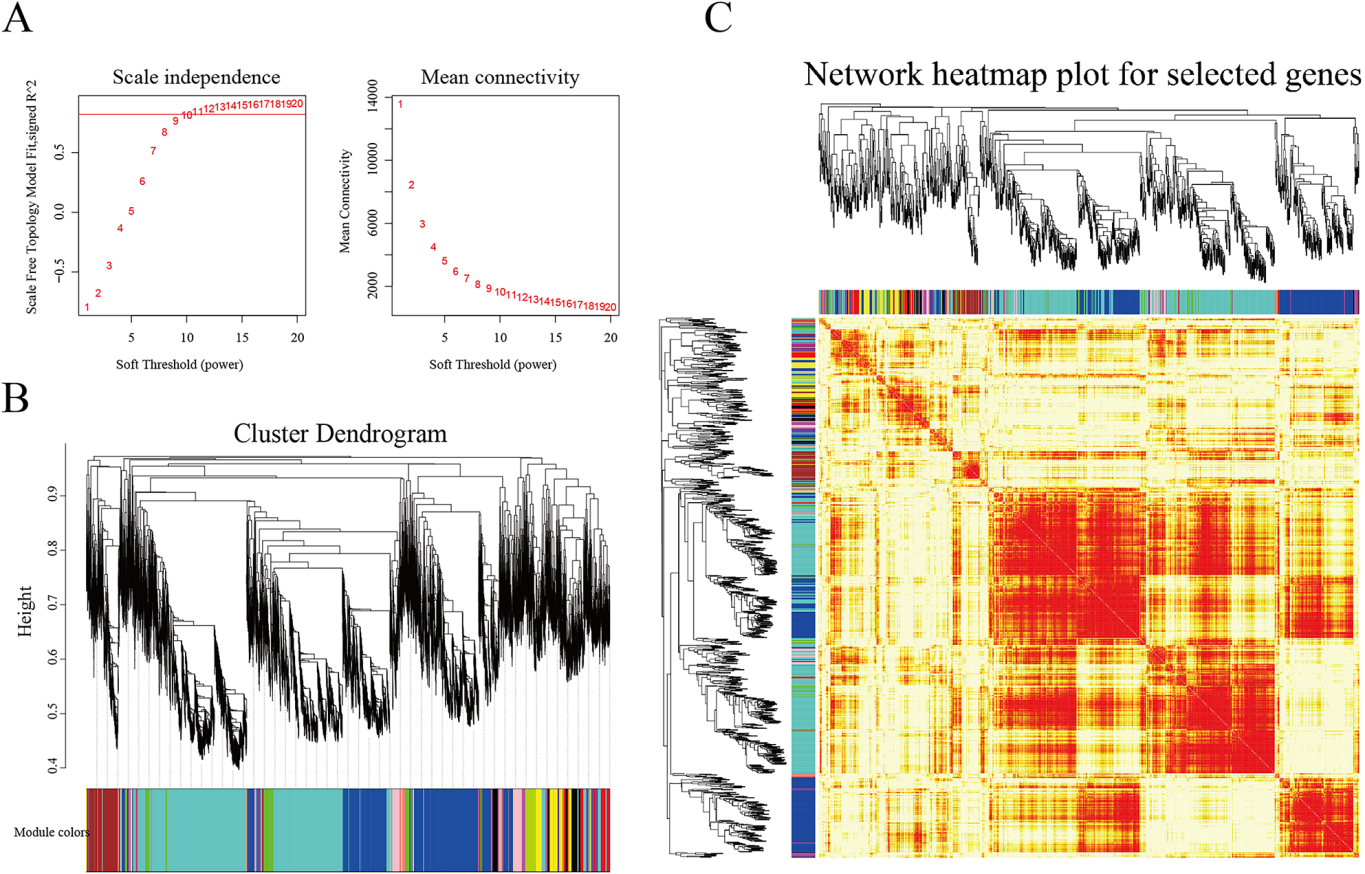
Sample clustering and gene module analysis. (**A**) Soft Threshold (power) selection diagram. The scaling-free topological model fit indices *R*^2^ corresponding to different soft thresholds are represented, at the red line by *R*^2^ = 0.8. When the power value is 20, the average connectivity tends to zero. (**B**) Module hierarchical clustering tree diagram. After obtaining different gene modules by dynamic shearing method, similar modules are merged to get the module division results. (**C**) Module gene clustering heat map.

**Fig. 4:**
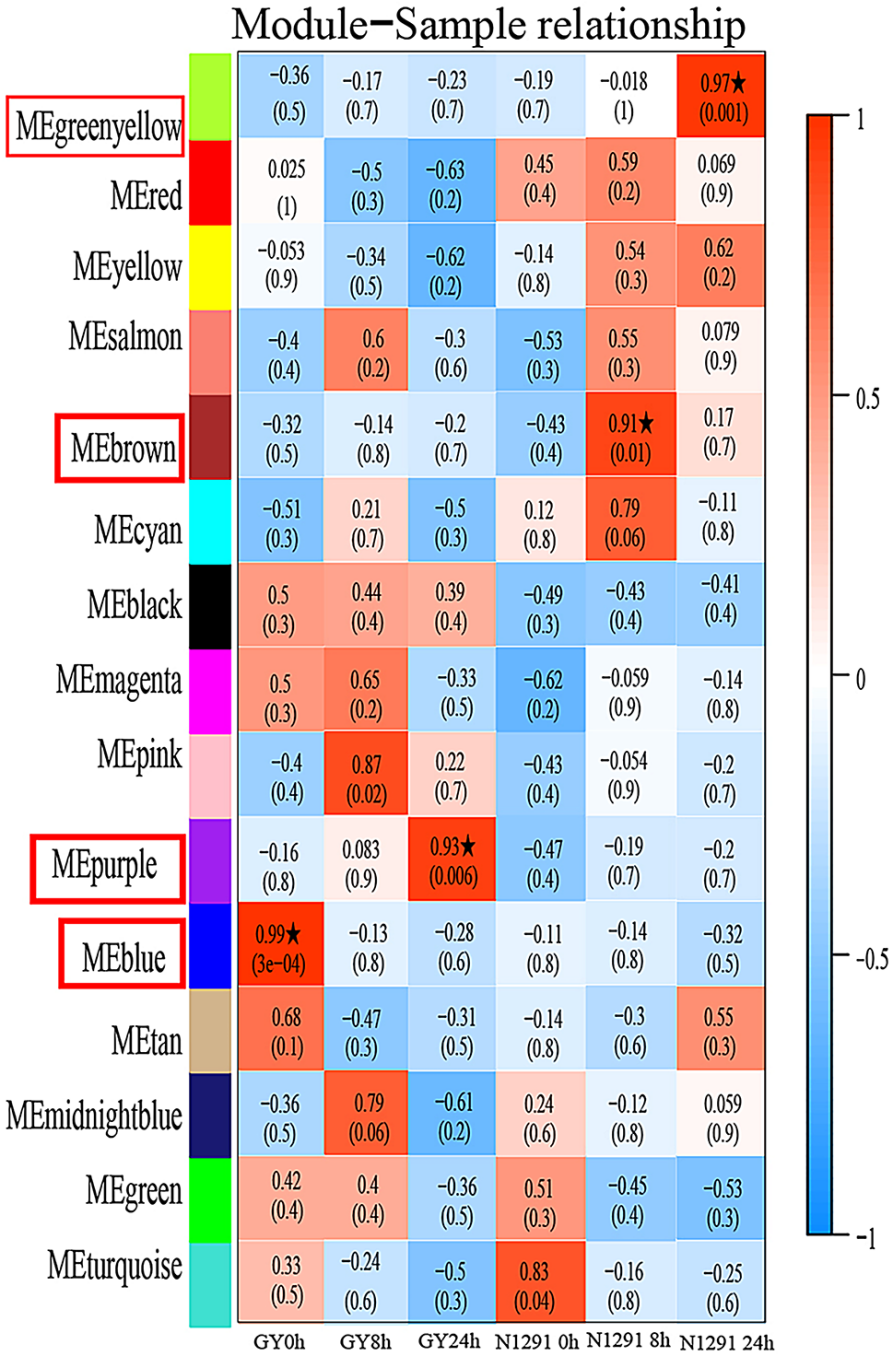
Module-sample correlation heat map. The horizontal coordinate is the sample, the vertical coordinate is the module, the first number in each box represents the correlation between the module and the sample, the number in parentheses represents the significance, and ME is the module attribute. The closer the correlation value is to ± 1, the stronger the positive/negative correlation between the module and the sample.

The WGCNA algorithm in the R programming environment (Version 3.5.0) was used to construct co-expressed networks. The results include Soft Threshold (power) selection diagram, Module hierarchical clustering tree diagram, Module gene clustering heat map (Fig. 3A-C), and Module-sample correlation heat map (Fig. 4). A total of 15 gene expression modules were constructed to determine the correlation between each gene module and each sample of *H. compressa* after waterlogging stress.

A heat map illustrating the module-sample correlations was constructed using information from each module and sample (Fig. 4). Four significantly correlated modules (*r*>|0.9|), blue, green-yellow, purple, and brown, were identified in *H. compressa* samples. Among them, the blue module was significantly associated with GY_0h (*r* = 0.99, *P* = 3e-04), the purple module was significantly associated with GY_24h (*r* = 0.93, *P* = 0.006), the brown module was significantly associated with N1291_8h (*r* = 0.91, *P* = 0.01), and the green-yellow module was significantly associated with N1291_24h (*r* = 0.97, *P* = 0.001). Notably, the blue module was strongly correlated with GY_0h, and its negative correlation with the other samples increased with the increase of waterlogging stress duration, which suggested that the genes in the blue module might have negative regulatory roles in GY under waterlogging stress. On the other hand, the green-yellow and purple modules were significantly positively correlated with N1291_24h and GY_24h, respectively. The positive correlation became increasingly significant as the duration of waterlogging stress increased, suggesting that the genes in the green-yellow and purple modules may have positive regulatory functions in N1291 and GY in response to waterlogging stress respectively.

### GO enrichment analysis of key gene modules

**Fig. 5:**
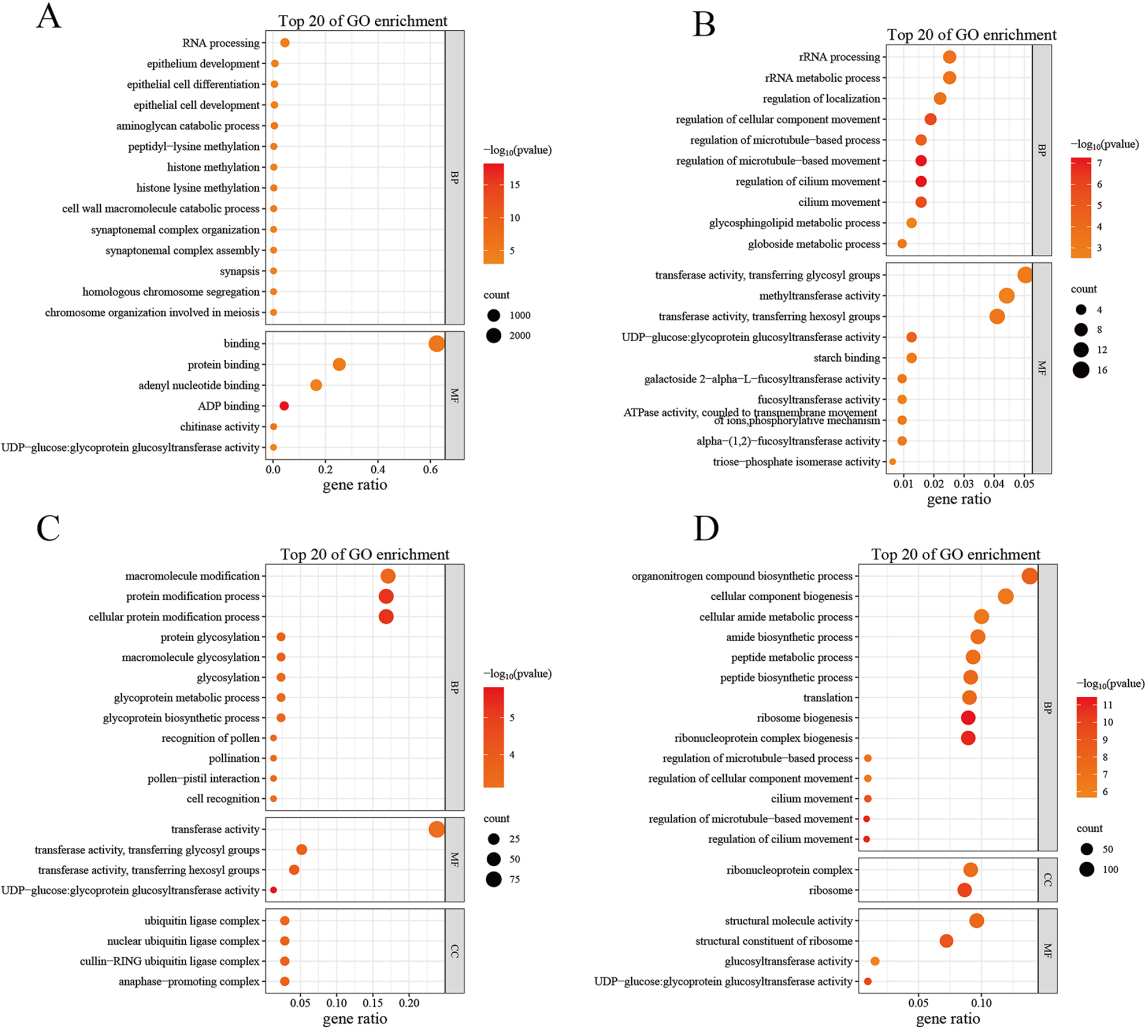
Key gene module GO enrichment analysis. (**A**-**D**) Indicate blue, green-yellow, purple, and brown modules respectively.

The GO functional enrichment of DEGs in the four modules was analyzed using clusterProfiler software to assess the enrichment of waterlogging stress tolerance-associated genes. The enriched genes were grouped in the biological process (BP), molecular function (MF), and cellular component (CC) categories. The top 20 GO terms that were significantly enriched (*P* < 0.05) in each module were plotted. Among them, a total of 2555 DEGs were analyzed in the blue module and analyzed for their GO categorization, with 14 terms enriched in BP and 6 terms enriched in MF among the top 20 GO terms (Fig. 5A). A total of 317 DEGs were analyzed in the green-yellow module and analyzed for their GO categorization, with 10 terms enriched in BP and 10 terms enriched in MF among the top 20 GO terms (Fig. 5B). A total of 386 DEGs were analyzed in the purple module, with 12 terms enriched in BP, 4 terms enriched in CC, and 4 terms enriched in MF among the top 20 GO terms (Fig. 5C). A total of 1039 DEGs were analyzed in the brown module, with 14 terms enriched in BP, 2 terms enriched in CC, and 4 terms enriched in MF among the top 20 GO terms (Fig. 5D).

By analyzing and comparing the 4 modules, it was apparent that all 4 modules were enriched to varying degrees in pathways related to energy metabolism, biosynthesis, and transferase activity. The blue module was enriched in biosynthesis and energy processes, such as ADP binding and aminoglycan catabolic processes. The green-yellow module was more significantly enriched in biosynthesis and transferase activities, such as regulation of cilia movement, UDP-glucose: glycoprotein glucosyltransferase activity, and regulation of cellular component movement. The most enriched pathways in the purple and brown modules were UDP-glucose: glycoprotein glucosyltransferase activity and ribosome biogenesis, respectively. Notably, UDP-glucose: glycoprotein glucosyltransferase activity was significantly enriched in the green-yellow, purple, and brown modules. Moreover, the ADP binding pathway was significantly enriched in the blue module (*P* < 0.01), related to ATP synthesis. In conclusion, it can be hypothesized, based on the GO enrichment analysis results, that enzyme synthesis and energy metabolism potentially play key regulatory roles in *H. compressa* in response to waterlogging stress.

### KEGG enrichment analysis of key gene modules

**Fig. 6:**
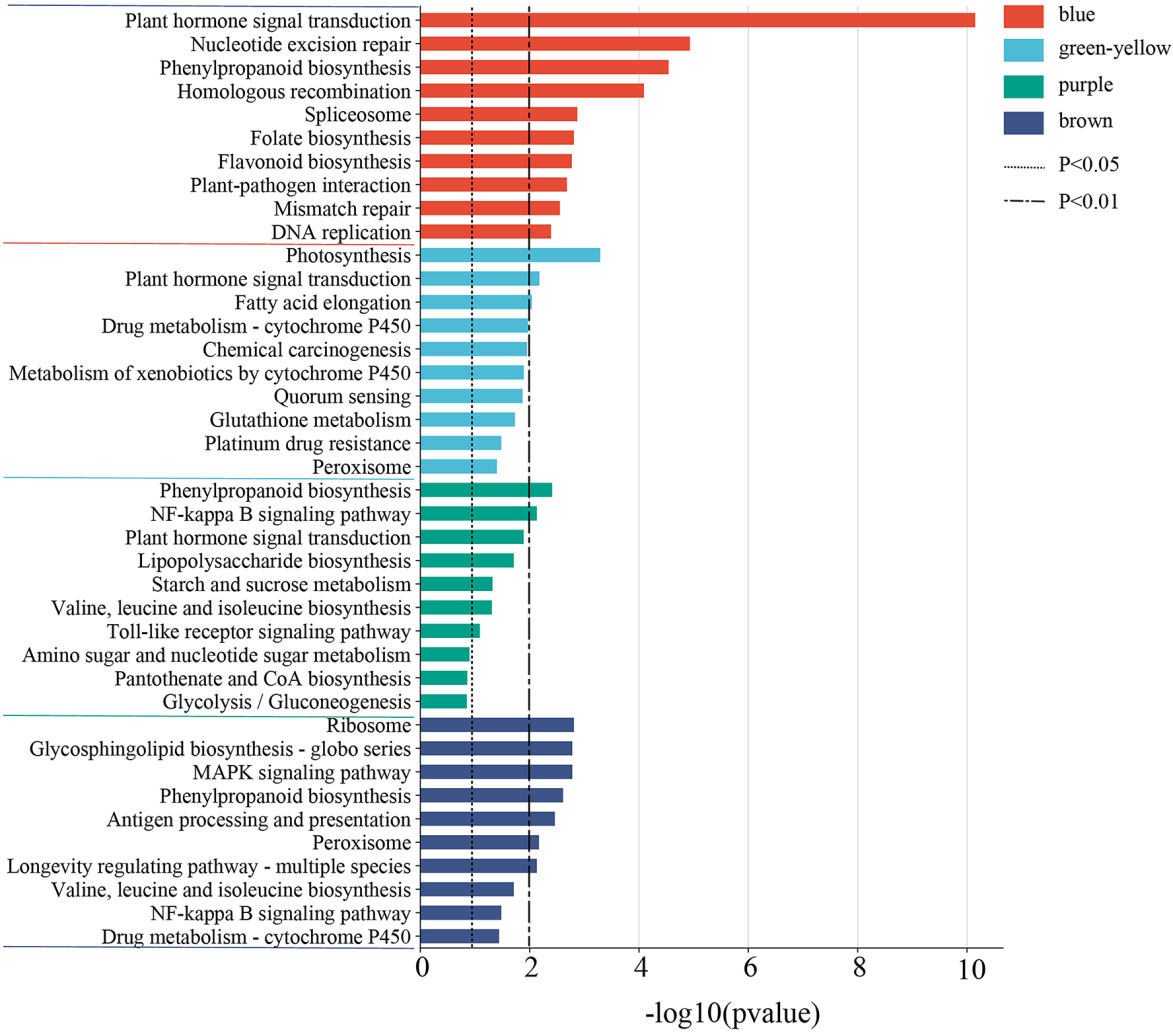
The top 10 KEGG enrichment pathways of key modules.

To further study the metabolic regulation pathways of co-expressed genes in key modules and explore the response mechanism of *H. compressa* with different waterlogging tolerance, KEGG pathway enrichment of DEGs in 4 modules was carried out in this study, and the top 10 pathways of each module were mapped (Fig. 6). Among them, the enrichment pathways in the blue module mainly involve Signal transduction, Genetic Information Processing, Biosynthesis of other secondary metabolites, etc. Among them, the first three significantly enriched (*P* < 0.01) pathways were Plant hormone signal transduction (*P* = 7.01E-11), Nucleotide excision repair, and Phenylpropanoid biosynthesis. The green-yellow module was mainly enriched in pathways such as metabolism and signal transduction. The top three significant enrichment pathways were Photosynthesis, Plant hormone signal transduction, and Fatty acid elongation. The first three enrichment pathways in the purple module were Phenylpropanoid biosynthesis, NF-kappa B signaling pathway, and Plant hormone signal transduction. Starch and sucrose metabolism were also significantly enriched in this module and participated in the Carbohydrate metabolism process. In the brown module, it was mainly enriched in Genetic Information Processing and biosynthesis and metabolism, and the most significant enrichment pathway was the Ribosome. Based on the comprehensive analysis of the four modules, KEGG pathway enrichment mainly involved Environmental Information Processing, Genetic Information Processing, biosynthesis and metabolism, and biosynthesis of other secondary metabolites. Plant hormone signal transduction, Starch and sucrose metabolism, Glutathione metabolism, Ribosome, and other pathways were significantly enriched, which may be closely related to the resistance and adaptation of *H. compressa* root system to waterlogging stress.

Plant hormone signal transduction (ko04075) was significantly enriched in the blue, green-yellow, and purple modules to varying degrees and, most significantly, in the blue module (*P* < 0.05). Plant hormone signaling is an important regulatory pathway in plants under abiotic stress. KEGG pathway analysis revealed that several signaling pathways associated with plant hormones, such as auxin (IAA), cytokinine (CK), abscisic acid (ABA), ET, gasmonic acid (GA), and brassinolide (BR) were activated in *H. compressa* when subjected to waterlogging stress (Fig. S2). In the blue module, most of the genes were found to be down-regulated in expression during treatment, but were more highly expressed in GY. These included ETR-encoding genes (*Cluster-38255.64854*, *Cluster-38255.92736*), which were down-regulated in GY and N1291 with the prolongation of waterlogging stress. Therefore, it was hypothesized that *H. compressa* can adapt to waterlogging stress by negatively regulating plant hormone such as ET (Fig. S2A).

Glutathione metabolism (ko00480) was significantly enriched in the green-yellow module (*P* < 0.05), and glutathione S-transferase (GST) encoding genes *Cluster-38255.63342* and *Cluster-38255.92747* were gradually up-regulated in N1291 as the duration of waterlogging increased. These findings suggest that the activation of GST-related genes may play an important role in the response of N1291 roots to waterlogging stress. In addition, an APX encoding gene, *Cluster-38255.92552*, was also up-regulated in N1291 and may act synergistically with GST to scavenge ROS generated during anaerobic respiration (Fig. S3A).

Starch and sucrose metabolism (ko00500) was significantly enriched in the purple module (*P* < 0.05), and most of the differentially expressed genes of this pathway were up-regulated in GY and N1291, and their up-regulation was more significant in GY. The genes of this pathway showed a trend of up-regulated expression with increasing duration of waterlogging stress in both GY and N1291. We hypothesized that GY and N1291 activated the starch and sucrose metabolism pathway genes to respond to waterlogging stress (Fig. S3B).

### Construction of a hub gene interaction network

**Fig. 7:**
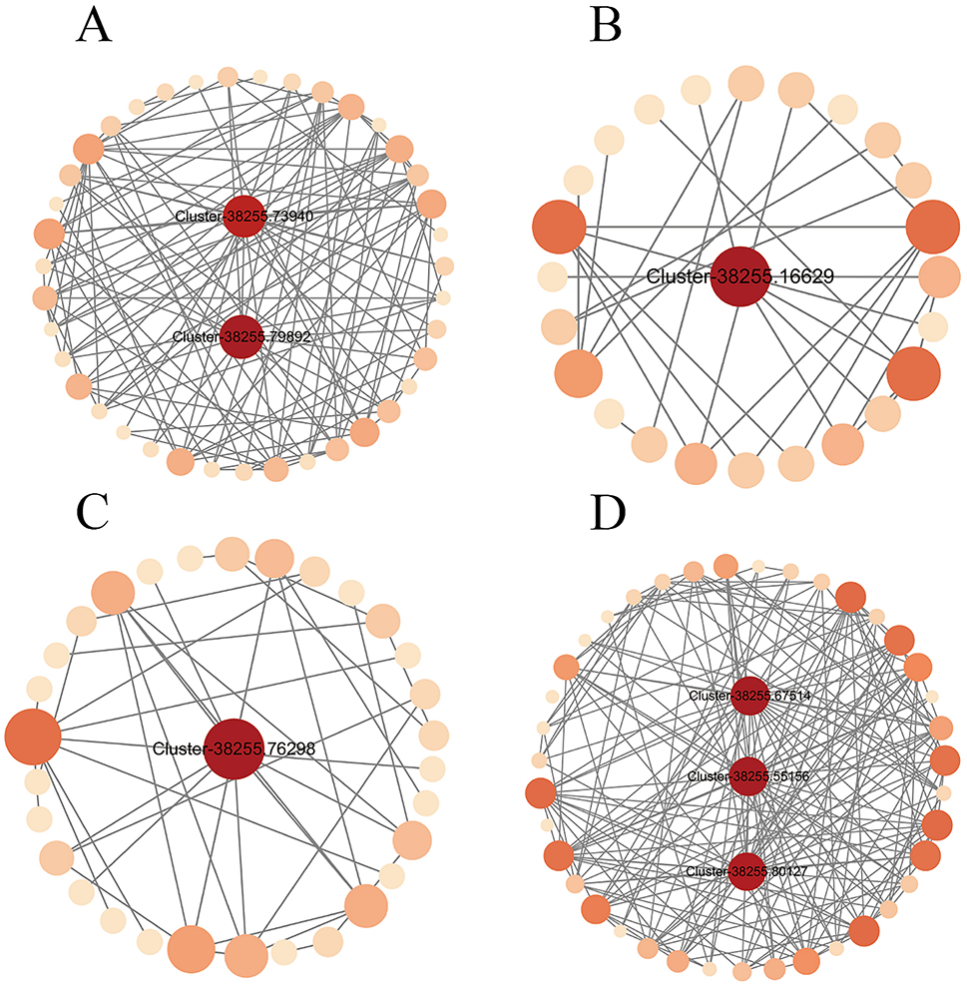
Interaction network analysis of key gene modules. (**A**-**D**) Interaction analysis of hub genes of the blue, green-yellow, purple and brown module respectively. In the middle is the hub gene, the darker the color and the larger the circle in the graph, the greater the degree value.

The genes linked to most genes within the WGCNA scale-free network modules, exhibiting high connectivity, correspond to hub genes, which generally possess key regulatory functions. Based on the correlation network diagram, we identified 7 hub genes in the 4 key modules. The hub genes for the blue module were *Cluster-38255.79892*, *Cluster-38255.73940* (Fig. 7A), for the green-yellow module, *Cluster-38255.16629* (Fig. 7B), for the purple module, *Cluster-38255.76298* (Fig. 7C), for the brown module, *Cluster-38255.67514*, *Cluster-38255.55156*, and *Cluster-38255.80127* (Fig. 7D).

The sequences of these 7 hub genes were aligned with the nucleotide sequences of *Arabidopsis thaliana* in the KOBAS 3.0 (http://bioinfo.org/kobas) online platform, and a function prediction of these genes was performed through the annotation of the homologous genes in *Arabidopsis thaliana*. Based on the results, 4 homologous genes were identified in *Arabidopsis thaliana*, *AtJ2* (*Cluster-38255.67514*), *TCTP* (*Cluster-38255.80127*), *NOP56* (*Cluster-38255.73940*), and *RACK1C_AT* (*Cluster- 38255.55156*). Three pathways are annotated to, ath03010 (Ribosomes), ath03008 (Ribosome biogenesis in eukaryotic cells) and ath04141 (Protein processing in the endoplasmic reticulum).Ribosomes are the site of translation of RNA into proteins, and ribosome activity is essential for protein synthesis and various metabolic activities in plants^40^. Based on the results of hub gene annotation, we hypothesized that gene transcription, translation, and protein synthesis may play a key role in *H. compressa* responses to waterlogging stress.

### Quantitative real-time PCR (qRT-PCR) validation of candidate core gene expression changes

**Fig. 8:**
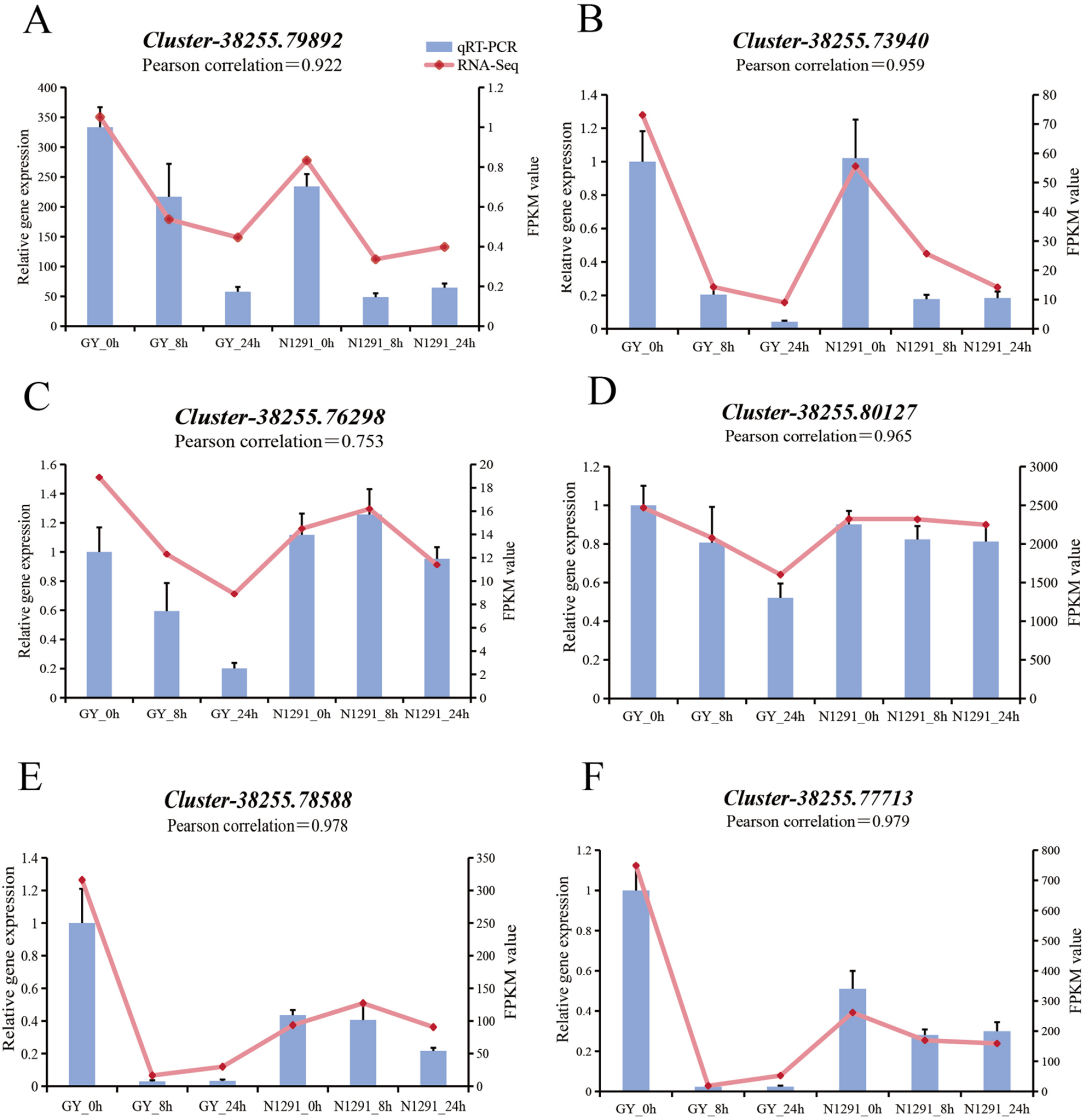
qRT-PCR validation results. Bar graphs indicate gene expression amounts and line graphs indicate FPKM value in RNA-seq data.

In order to verify the accuracy and reproducibility of the transcriptome analysis results, the expression of 4 hub genes and 2 differentially expressed genes in key pathways were randomly selected and verified by qRT-PCR analysis. The results showed a high correlation between the expression of the assessed genes and RNA-Seq, with a pearson correlation coefficient > 0.9, except for *Cluster-38255.76298*. It indicates that the gene expression trend identified by qRT-PCR is essentially by the FPKM results of individual genes in the transcriptome analysis and that the RNA-Seq data were reliable (Fig. 8).

## Discussion

### Regulation of antioxidant enzymes under waterlogging stress

Under normal conditions, ROS in plant cells are in dynamic equilibrium and are maintained at low levels by antioxidant enzymes to prevent lasting damage to plant cells^41^. In this study, the activities of SOD and POD in GY and N1291 roots were higher than those in the control group after 8 h and 24 h of waterlogging treatment. The SOD activity of GY and N1291 was significantly higher than that of the control group (*P* < 0.05), and the SOD activity of GY was higher than that of N1291. This is similar to *Solanum lycopersicum*^42^, *Triarrhena sacchariflora*^43^. The increase of antioxidant enzyme activity is a key strategy for plants to effectively scavenge ROS and resist waterlogging stress^44^. When *H. compressa* was damaged by waterlogging stress, the antioxidant enzyme system could respond quickly in the short term and alleviate the adverse effects of waterlogging stress on *H. compressa*.

### Regulation of glutathione metabolism pathway under waterlogging stress

In the green-yellow module, Glutathione metabolism (ko00480) is significantly annotated. The results of gene clustering showed that GST and APX coding genes were up-regulated in N1291 after waterlogging treatment. GSH is the major intracellular antioxidant in many organisms and protects cells from various free radicals such as ROS, lipid peroxides, exogenous toxins, and heavy metals^45,46^. GST is the key enzyme that catalyzes the initial step in glutathione conjugation with various substrates^47^. Studies have shown that GST-encoding genes are significantly up-regulated in response to waterlogging stress in plants^48^. APX, a key enzyme involved in the AsA-GSH cycle, enhances plant ROS scavenging and resistance to oxidative stress and plays an important role in the maintenance of cellular redox homeostasis^49,50^. As demonstrated by Yao et al.^51^, exogenous GSH treatment could reduce the extent of damage in *Capsicum annuum* fruits during cold storage by activating the AsA-GSH cycle and increasing their antioxidant capacity. Chen et al.^52^ reported that the APX activity of *Pinellia ternata* increased under drought stress, the content of AsA and GSH increased, and the AsA-GSH cycle was enhanced, thereby reducing the accumulation of ROS. Combined with the results of physiological experiments, it can be seen that in addition to coping with oxidative stress through antioxidant enzymes such as SOD and POD, *H. compressa* may also resists waterlogging oxidative damage by the AsA-GSH cycle and ultimately reduces ROS accumulation.

### Regulation of plant hormone signal transduction pathway under waterlogging stress

Plant hormones regulate normal plant growth and developmental processes, seed germination, root elongation, and other processes by regulating gene expression, protein translation, and metabolic pathways, and play a key role in the responses to abiotic stresses such as drought, waterlogging, etc^53–55^. This study found that ET, IAA, ABA, and BR hormone signaling pathways were significantly enriched in the blue module; the IAA, ABA hormone signaling pathways were significantly enriched in the green-yellow module; The IAA, ET, ABA and JA hormone signaling pathways were significantly enriched in the purple module. This indicates that *H.compressa* is induced to produce a cultivars of plant hormones at 8 h and 24 h of waterlogging, which is involved in the regulation of waterlogging stress. In particular, Plant hormone signal transduction was significantly enriched in the blue module. Through gene clustering enrichment, it was found that the ETR coding genes *Cluster-38255.64854* and *Cluster-38255.92736*, AUX/IAA coding genes *Cluster-38255.85718* and *Cluster-38255.75691* in the blue module were gradually down-regulated with the prolongation of *H.compressa*waterlogging time, showing a negative regulatory trend. ET can interact with IAA, GA, ABA and other hormones, and participate in the response and adaptation of plants to hypoxic conditions to improve waterlogging stress tolerance^56^. For example, ET regulates root development and elongation by changing IAA biosynthesis, transport and signal transduction^57^. In this study, the DEGs of Plant hormone signal transduction were mostly down-regulated. Similar results were found in other plants. For example, Xiong et al.^58^ found that the *submergence-1* ( *SUB1* ) gene of rice inhibited the production of ET under waterlogging stress, thereby inhibiting the production of GA and the sensitivity of cells to GA, and prolonging the survival time of *Oryza sativa* by reducing energy consumption and inhibiting growth. Liu et al.^59^ discovered that lowering the ET content in *Gossypium hirsutum* attenuated hypoxic damage and the inhibition of growth and development of *Gossypium hirsutum* under waterlogging stress. The current study speculates that *H. compressa* can improve the ability of *H.compressa* to resist waterlogging stress by reducing the production of plant hormones such as ET and IAA, regulating energy metabolism and reducing the damage of ROS to the cell membranes.

### Regulation of starch and sucrose metabolic pathway under waterlogging stress

Usually, plants can reduce the adverse effects of waterlogging stress through energy-saving metabolism and antioxidant defense systems, and this series of reactions are regulated by differential genes^60^. Enhances waterlogging tolerance by activating carbon metabolism-related pathways to provide energy and inhibiting partially energy-consuming biosynthetic pathways^61^. In this study, DEGs in key modules were significantly enriched in carbon metabolism-related pathways such as Starch and sucrose metabolism, Galactose metabolism, Glycolysis/Gluconeogenesis and Phenylpropanoid biosynthesis. Carbohydrates are an important source of energy metabolism in plants. Zeng et al.^62^ used transcriptome sequencing technology to analyze the leaf data of *Medicago sativa* and found that the Starch and sucrose metabolism pathway was activated after waterlogging stress to respond to waterlogging stress. When waterlogging stress was received, *H. compressa* responded rapidly to carbohydrate metabolism-related genes, regulated the response process of *H. compressa* to waterlogging stress, and improved the waterlogging resistance of *H. compressa*. In addition, Starch and sucrose metabolism were significantly annotated to the purple module, while the purple module was significantly associated with GY_24h. Differential gene clustering showed that the coding genes of 6-phosphate trehalose (T6P), β-glucosidase (BGLU) and β-Amylase related to sucrose content were up-regulated after 8 h and 24 h of waterlogging stress. Zeng et al.^63^ found that the contents of soluble sugar and sucrose in leaves of *Arachis hypogaea* increased after 5 d and 10 d of waterlogging stress, and the genes related to sucrose decomposition, sucrose synthase 4 (SS) and BGLU11, were up-regulated, while the genes related to starch synthesis were down-regulated, which was similar to the results of this experiment. In this study, when *H. compressa* was subjected to waterlogging stress, the coding genes of T6P (*Cluster-38255.54401*, *Cluster-38255.73736*), BGLU (*Cluster-38255.73736*) and β-Amylase (*Cluster-38255.118361*) were significantly up-regulated, accelerating the decomposition of sugar, providing substrates for glycolysis to produce ATP and saving carbohydrates, maintaining the energy supply of *H. compressa*. It is worth noting that although the DEGs of the Starch and sucrose metabolism pathway were up-regulated in both GY and N1291, the expression level in GY was significantly higher than that in N1291, which also indicated that the hypoxia and energy regulation mechanism of GY was stronger than that of N1291 in the face of waterlogging stress. This is also one of the characteristics of GY as a waterlogging-tolerant cultivars compared with N1291. Similarly, some scholars ' research also confirms this view. The results of Shen et al.^64^ showed that the differences in waterlogging tolerance of wheat were closely related to glycolysis, starch and sucrose metabolic pathways. Genes related to glycolysis, starch and sucrose metabolism in embryos and endosperm of waterlogging-tolerant wheat (BN607) could be rapidly induced to express. Zeng et al.^62^ found that the coding gene *Cluster-1252.44338* of β-Amylase was induced to express in the waterlogging-resistant* Medicago sativa* cultivar M12, which was 2.8 times higher than that in the control group, while the expression level in the waterlogging-sensitive cultivar M25 did not change significantly. In this study, the same results were obtained. The expression of *Cluster-38255.118361*, the encoding gene of β-Amylase in GY, was 1.15 and 2.46 times higher than that of 0 h at 8 h and 24 h, respectively, while there was no significant change in N1291. The above results indicate that the Starch and sucrose metabolism pathway is an effective strategy for *H. compressa* to resist waterlogging stress and can promote the energy metabolism homeostasis of *H. compressa* roots. The change of DEGs in this pathway in GY was more significant than that in N1291, which may be one of the characteristics of GY as a waterlogging-tolerant cultivar.

### Regulation of functional analysis of hub genes and ribosome pathway under waterlogging stress

Abiotic stress-induced gene expression in plants underlies adaptation to complex environmental changes^65^. mRNA translation into proteins enables plants to quickly adjust and adapt to environmental changes^66^. Seven hub genes related to waterlogging tolerance were preliminarily mined by WGCNA analysis. Compared with *Arabidopsis thaliana* gene annotation, four homologous genes such as *AtJ2* (*Cluster-38255.67514*), *AtTCTP* (*Cluster-38255.80127*) and *RACK1C_AT* (*Cluster-38255.55156*) were found. KEGG enrichment analysis showed that most hub genes were annotated to Ribosome, Ribosome biosynthesis and other related pathways. Involved in ribosome biosynthesis and reaction. Other hub genes that could not be annotated may be species-specific genes that can be further explored in subsequent studies. *AtJ2*, a molecular chaperone in *Arabidopsis thaliana*, is involved in responses to various abiotic stresses and can help stabilize intracellular protein structures and prevent protein denaturation, thereby enhancing plant resistance to oxidative stress^67,68^. Studies have shown that *AtJ2* is involved in the regulation of water stress response in *Arabidopsis thaliana*^69^. Translationally controlled tumor protein (TCTP) controls growth by regulating the G1/S transition in cell cycle progression and plays a role in growth regulation, stress signaling, and programmed cell death (PCD)^70–72^. Chen et al.^73^ found that TCTP may be a potential PCD regulator in *Zea mays *under waterlogging stress. The formation of aerenchyma by PCD enhances root gas exchange, so that plants can tolerate hypoxia caused by prolonged waterlogging^74,75^. Therefore, the hub gene *Cluster-38255.80127* may also be related to PCD in *H. compressa*. In future studies, the changes in ARs and aerenchyma in *H. compressa* roots can be a concern. And the role of *Cluster-38255.80127* in root morphological changes.

**Fig. 9:**
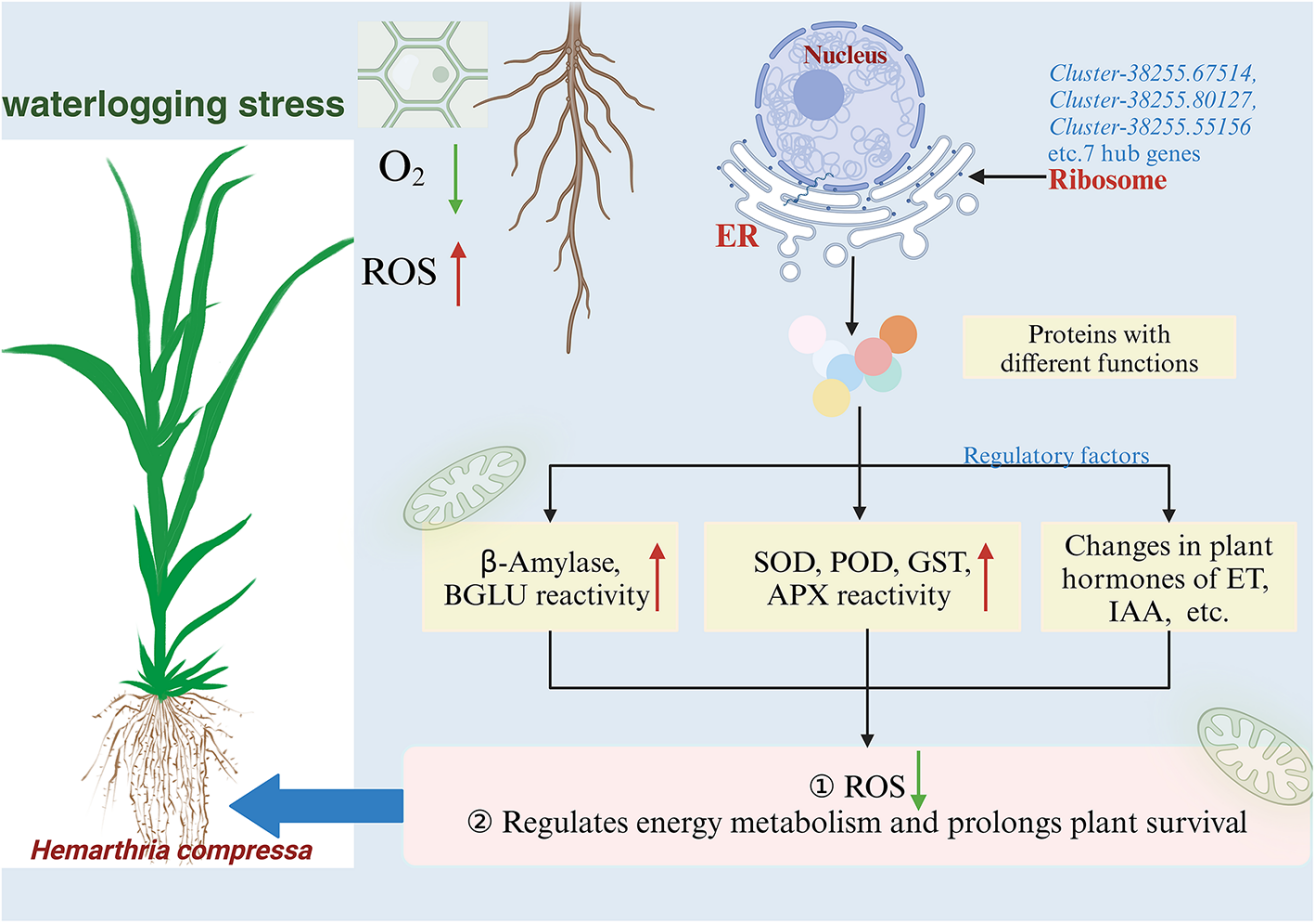
Model diagram of *H. compressa* response mechanism under waterlogging stress. The arrows in the figure indicate the change of content or activity. Upward red arrows indicate an increase, while the downward green arrows indicate a decrease. The pink box indicates the changes in ROS content and energy regulation in *H. compressa* roots after response.

Ribosome function is essential for the production and regulation of proteins and enzymes in plants under abiotic stress, and this coordinated process is tightly regulated by gene expression^40^. Zhang et al.^76^ found that the synthesis of ribosomal proteins during germination under waterlogging stress was higher in high-vigor *Zea mays* seeds than in low-vigor *Zea mays* seeds, as well as the POD, SOD, and APX activities. In *Arabidopsis thaliana*, ribosome mutants exhibited significant IAA-deficient phenotypic traits, such as root growth retardation and plant dwarfism^77^. Ribosomes serve as the first step in regulating protein synthesis and downstream metabolic compounds. We therefore hypothesize that *H. compressa* promotes a series of pathways related to protein synthesis under waterlogging stress by inducing gene expression, facilitating transcription, translation, and protein processing. This contributes to the synthesis and activation of enzymes or other response factors of a wide range of regulatory mechanisms, such as antioxidant defense, energy metabolism and plant hormone, to regulate metabolic activities in vivo, enhancing *H. compressa* waterlogging stress tolerance. This study preliminarily constructed a simple response model to explain the response mechanism of ribosomes and other systems of *H. compressa* under submergence stress. In the future, it is necessary to deeply analyze its function and mechanism (Fig. 9).

## Conclusion

In this study, the physiological and molecular changes of *H. compressa* roots after waterlogging stress were compared and analyzed. The results showed that waterlogging stress triggered the antioxidant enzyme system of *H. compressa*, and the activities of SOD and POD were significantly enhanced. The module-sample correlation heat map was constructed by WGCNA analysis, and 4 key modules significantly positively correlated with waterlogging stress samples were obtained. The functional enrichment analysis of key module DEGs showed that pathways such as Starch and sucrose metabolism, Plant hormone signal transduction, Ribosome and Glutathione metabolism were important regulatory pathways for *H. compressa* to respond to waterlogging stress. The Starch and sucrose metabolism pathway in GY was more significant than that in N1291, indicating that GY was more resistant to waterlogging than N1291. Seven hub genes related to waterlogging stress were identified, most of which were annotated to Ribosome, Ribosome biosynthesis and other related pathways. Homologous genes such as *AtJ2* and *AtTCTP* were compared with *Arabidopsis thaliana* gene annotation. It may be involved in the regulation of the PCD process in *H. compressa*, forming aerenchyma to strengthen root gas exchange to alleviate waterlogging damage. This study preliminarily excavated the molecular mechanism and hub gene of *H. compressa* root response to submerged stress, and provided a certain direction for the future study of *H. compressa* waterlogging tolerance genes. In the future, we can further study the role of ribosome and PCD in the waterlogging resistance of *H. compressa*, or further analyze the function of the hub gene through molecular marker-assisted selection or gene function verification, so as to help screen waterlogging-tolerant cultivars and improve crop yield.

## Methods

### Plant material growth conditions and waterlogging stress treatment

The materials used in this experiment were two *H. compressa* cultivars GY (from Sichuan Agricultural University) and N1291 (N1201801291, from Southwest University), which were screened by our group in the early stages of the experiment and which differed greatly in waterlogging stress tolerance. The difference evaluation of waterlogging tolerance between GY and N1291 was identified by the members of the research group based on the analysis of morphological, microscopic, and physiological indexes related to waterlogging stress (the study has not been published). The number of ARs, root number, root volume, leaf length, leaf width and plant height were measured by morphological changes. The root activity, relative conductivity, total chlorophyll content, malondialdehyde, transpiration rate and soluble protein content were measured by physiological indexes. Microstructure observation showed that the reduction of vessel number, vascular cross-sectional area and cortical parenchyma thickness of GY was significantly less than that of N1291. Based on the above indicators, the scores were evaluated, and the waterlogging-tolerant variety GY and the non-waterlogging-tolerant type N1291 were selected. In the current study, the healthy and disease-free rhizome of *H. compressa* was selected as the test plant. *H. compressa* plants were planted in pots (15.0 cm in caliber, 13.5 cm in height, with a drainage hole of about 2.0 cm in diameter at the bottom), and the test soil was nutrient soil, vermiculite and perlite (3:1:1, V/V/V). Six plants were planted in each pot, and 40 pots were planted for each cultivar. After transplanting, they were placed into an artificial photosynthetic incubator in the pasture laboratory of Rongchang Campus of Southwest University of China. The temperature was set at 23/16 ℃ (day/night), the relative humidity was 80%, the light intensity was 5000 lx, and the photoperiod was 16/8 h (day/night). During cultivation, the plants were watered every 3 d, and 1/2 Hoagland nutrient solution (Qingdao Haibo Biotechnology Co., Ltd.) was applied once a week. The growth of *H. compressa* was observed periodically, and the plants were subjected to waterlogging stress at the tillering stage. Well-established and uniform *H. compressa* plants were selected and placed in a water tank. Water was slowly added into the tank until it was 2–3 cm above the highest point of the plant, and the water level was observed every day to ensure that the highest point of the *H. compressa* plant was submerged. With 0 h assigned as the control group, physiological and biochemical assessment was performed, and root samples for transcriptome sequencing were taken after 8 h and 24 h of waterlogging stress (mixed sampling method, three biological replicates per group). All fresh root samples were frozen in liquid nitrogen and stored at −80 °C immediately after sampling until physiological indicators were measured and transcriptome sequencing was performed.

### Measurement of SOD and POD activities

SOD and POD activities of *H. compressa* root samples were determined after 0 h, 8 h, and 24 h of waterlogging stress. SOD activity was assessed by the nitrogen blue tetrazolium (NBT) colorimetric assay, and POD activity was assessed using the guaiacol assay. The sample extraction and assay methods have been described in Zhang et al.^78^.

### RNA extraction and transcriptome sequencing

RNA was extracted from 18 *H. compressa *root samples using the Tiangen RNAprep Pure Plant Plus Kit (Polysaccharides & Polyphenolics-rich). RNA integrity was assessed using an Agilent 2100 bioanalyzer^79^. After the quality control of the samples, library construction was carried out according to the general NEB library construction method. Subsequently, library quality testing was carried out. Firstly, preliminary quantification was performed using the Qubit2.0 Fluorometer, and the library was diluted to 1.5 ng/µL. Then, the Agilent 2100 bioanalyzer was used to detect the insert size in the library, and finally, qRT-PCR was used to accurately quantify the effective concentration of the library (which was higher than 2 nM). The RNA-seq library was sequenced on the Illumina platform to produce 150 bp PE raw reads. High-quality clean reads were obtained after filtering low-quality reads and N-containing reads. The Error rate, Q20, Q30 and GC content of clean reads were calculated. The sequencing and calculation process was completed by Novogene Bioinformatics Technology Co., Ltd. (Beijing, China).

### *De Novo* transcriptome assembly and differential gene expression

Currently, *H. compressa* has no reference genome. Therefore, this study performed *de novo *assembly of clean reads to generate a reference sequence. To address potential splicing variations, Trinity^80^ was used for *de novo *assembly, and the resulting unigenes were utilized as the reference sequence. Transcript levels were quantified using RSEM^81^(default settings), and clean reads were aligned to the reference sequence using Bowtie (Table S2). Differential gene expression analysis was performed using the R package DESeq2^82^ (http://www.bioconductor.org/packages/release/bioc/html/DESeq2.html). The threshold was |log_2_ (fold-change)| > 1.5 and padj < 0.05.

### Weighted gene co-expression network construction and visualization analysis

The R (Version 3.5.0)^83^ WGCNA package was used to construct the co-expression networkand (https://cran.r-project.org/*).* The average expression level of DEGs at different time points was calculated, and DEGs with expression levels lower than 5 were filtered out. Finally, 10,505 DEGs were used to construct a co-expression gene matrix and input into the algorithm. The default automatic network construction function blockwiseModules is used to construct the co-expression module, β = 10 (*R*^2^ > 0.8), “TOMType = unsigned, minModuleSize = 30, reassignThreshold = 0, mergeCutHeight = 0.25”. The relationship between each network module and the sample phenotype was analyzed, and the modules with significant Pearson > 0.9 were selected. GO (https://geneontology.org) functional enrichment and KEGG^84–86^ (https://www.kegg.jp) pathway enrichment analyses were performed on the set of differential genes in the screened modules, and significantly enriched pathways were identified at *P* < 0.05. Interaction networks of proteins encoded by the differentially expressed genes were assessed using the STRING protein interaction database (https://cn.string-db.org). Finally, the key module network was mapped in Cytoscape (3.10.2) (https://Cytoscape.org) software. The degree values of the module genes were calculated using the CytoCNA plugin and based on the degree values of the hub genes.

### Quantitative real-time PCR analysis

The quality of transcriptome sequencing was verified by qRT-PCR analysis. The 18 S gene was used as an internal reference gene (primer sequence content is shown in Table S3), and 4 hub genes and 2 DEGs in key pathways were randomly selected for expression validation. A reverse transcription kit (TUREscript 1st Stand cDNA SYNTHESIS Kit) from Aidlab was used, and reverse transcription was performed according to the kit instructions. The reverse transcription reaction conditions are as follows, the reaction was carried out at 45 °C for 40 min, and then at 65 °C for 10 min. After the reaction, the obtained cDNA was stored at −80 °C. qRT-PCR was performed using a qTOWER 2.2 (Analytik Jena) model fluorescence quantitative PCR instrument (Germany). Amplification at 95 °C for 3 min. The cycle reaction is 95 °C 10 s denaturation, 60 °C 30 s annealing and extension, set up 40 cycles, and then analyze the melting curve. Relative gene expression levels were calculated by the 2^−ΔΔCt ^method^87^.

### Statistical analysis

Data statistics were performed using Microsoft Office 2021, and data were further analyzed using IBM SPSS Statistics 27 software. The significance of differences between treatments was analyzed using ANOVA (*P* < 0.05). On the Novogene Cloud platform (https://magic.novogene.com/), the screening of differentially expressed genes and the analysis of the Venn diagram were carried out, and the mapping and data visualization were carried out using the relevant R package. Drawing with Origin 2018, Adobe Illustrator 2024, and Biorender (https://app.biorender.com/).

### Plant ethics statement

In this study, the plant materials were GY and N1291 *H. compressa*. GY is derived from Sichuan Agricultural University, and passed the validation of Ministry of Agriculture and Rural Affairs of the People’s Republic of China in July 1987. No.011. N1291 was collected from the forage experimental base of Southwest University, and was collected and identified by Professor Bing Zeng, a forage research expert of Southwest University. It has not been stored in the public specimen bank. We declare that the collection and experimental research of plant materials in this study are permitted by the relevant units and in accordance with the relevant institutions, national and international regulations. The collection and preservation of plant cultivation and plant materials are operated in accordance with the “Methods” section.

## Electronic supplementary material

Below is the link to the electronic supplementary material.

Supplementary Material 1

## Data Availability

The sequencing data were submitted to NCBI SRA database with the accession number PRJNA1053919.
