# Supplementary material for: WGCNA analysis reveals hub genes in the Hemarthria compressa roots in response to waterlogging stress
Source: Sci Rep. 2025 Apr 22;15:13841. doi: 10.1038/s41598-025-94873-7 (PMC12015520; doi:10.1038/s41598-025-94873-7)
Supplement: Supplementary file 1 — Supplementary Material 1 [file 41598_2025_94873_MOESM1_ESM.docx]

| **Supplementary table 1. Transcript assembly statistics** | | | | | | | | |
| --- | --- | --- | --- | --- | --- | --- | --- | --- |
| **Index** | **300-500bp** | **500-1kbp** | **1k-2kbp** | **>2kbp** | **Total** | **Median length(bp)** | **N50(bp)** | **N90(bp)** |
| Transcripts | 177717 | 152612 | 109751 | 64059 | 504139 | 678 | 1515 | 446 |
| Unigenes | 112017 | 81872 | 42090 | 20571 | 256550 | 553 | 1138 | 396 |

| **Supplementary table 2. Comparison results of sample reference sequence** | | |
| --- | --- | --- |
| **Sample name** | **Total reads** | **Total mapped** |
| GY_0h_1 | 44482226 | 31685042 |
| GY_0h_2 | 46384678 | 33449188 |
| GY_0h_3 | 41467766 | 30709904 |
| GY_8h_1 | 45446066 | 33460710 |
| GY_8h_2 | 44333420 | 31662176 |
| GY_8h_3 | 43804784 | 32052142 |
| GY_24h_1 | 48054340 | 34892538 |
| GY_24h_2 | 43132762 | 32795358 |
| GY_24h_3 | 38394502 | 28438340 |
| N1291_0h_1 | 46323968 | 32922246 |
| N1291_0h_2 | 44621484 | 31767904 |
| N1291_0h_3 | 41600418 | 29314678 |
| N1291_8h_1 | 40323264 | 29694464 |
| N1291_8h_2 | 44611954 | 32866100 |
| N1291_8h_3 | 43129978 | 31294452 |
| N1291_24h_1 | 41255826 | 30161576 |
| N1291_24h_2 | 41725728 | 30717430 |
| N1291_24h_3 | 47594890 | 34672506 |

**Supplementary table 3. List of target gene primers**

| **Gene ID** | **Forward primer（5'-3'）** | **Reverse primer（5'-3'）** |
| --- | --- | --- |
| *18S*（internal reference） | CAACCATAAACGATGCCGA | AGCCTTGCGACCATACTCC |
| *Cluster-38255.79892* | TTCCAAGAAGCGGCACTG | GCAACCAATCATCAGAGGACTT |
| *Cluster-38255.73940* | CGATGCTGAAGAGACTGA | GGATTCATACGCCATACAAC |
| *Cluster-38255.76298* | ACCCAGAGAAGCGTGAGATA | TTCCACCACCTCCTCCAA |
| *Cluster-38255.80127* | GGCAGTGAGGTTCTTGATGTA | TGGTGATGATGAGGGTGTTG |
| *Cluster-38255.78588* | GGTGTGGAACGACAACGA | CTTCTCGATCAGGTGGTAGTC |
| *Cluster-38255.77713* | CTACTCCGACGACAAGAT | GGCAGCATCAGGTAATTC |

**
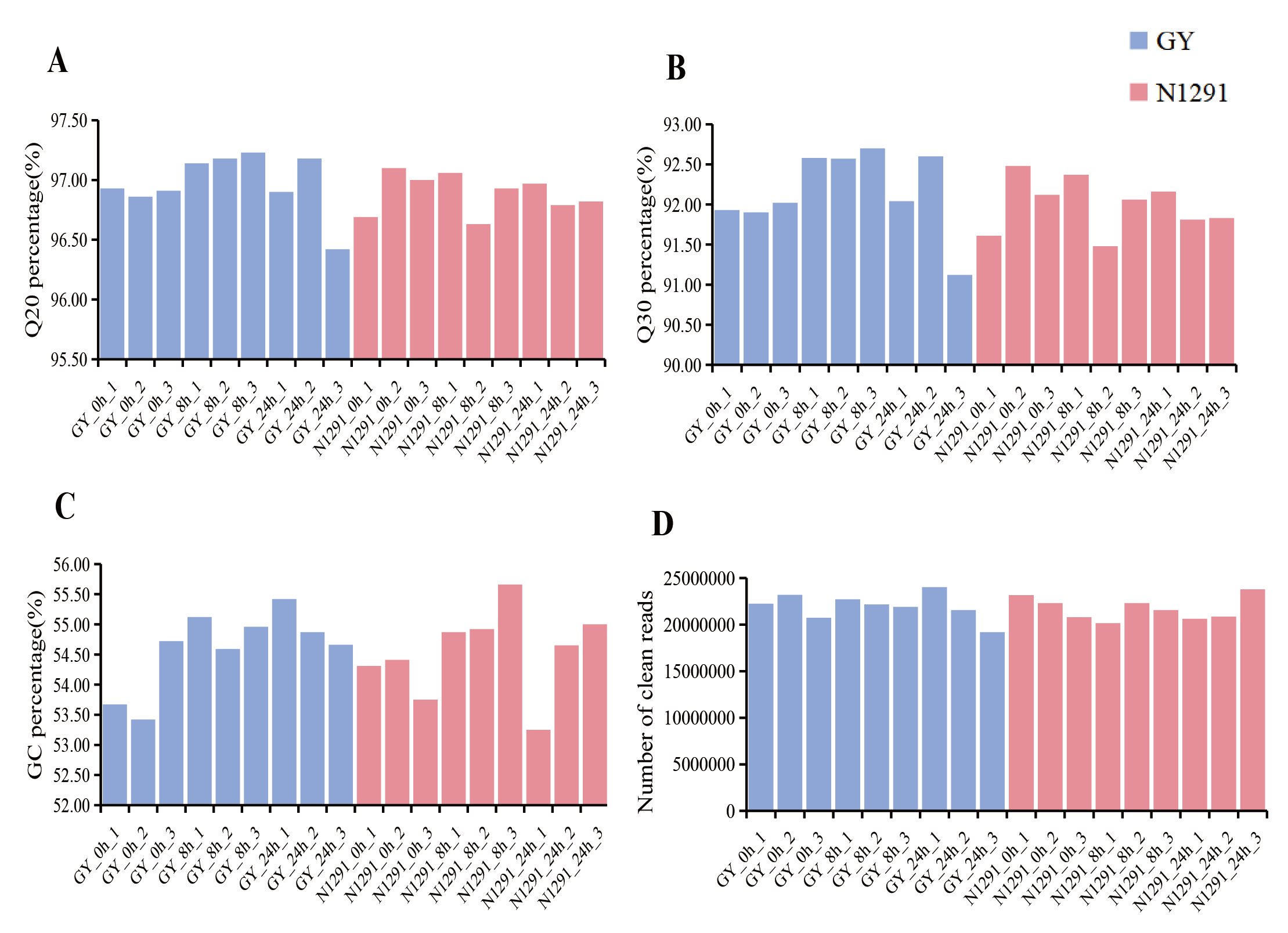
**

**Supplementary figure 1**. Sample sequencing quality results. (A) Q20 percentage; (B) Q30 percentage; (C) GC content percentage; (C) Number of clean reads.


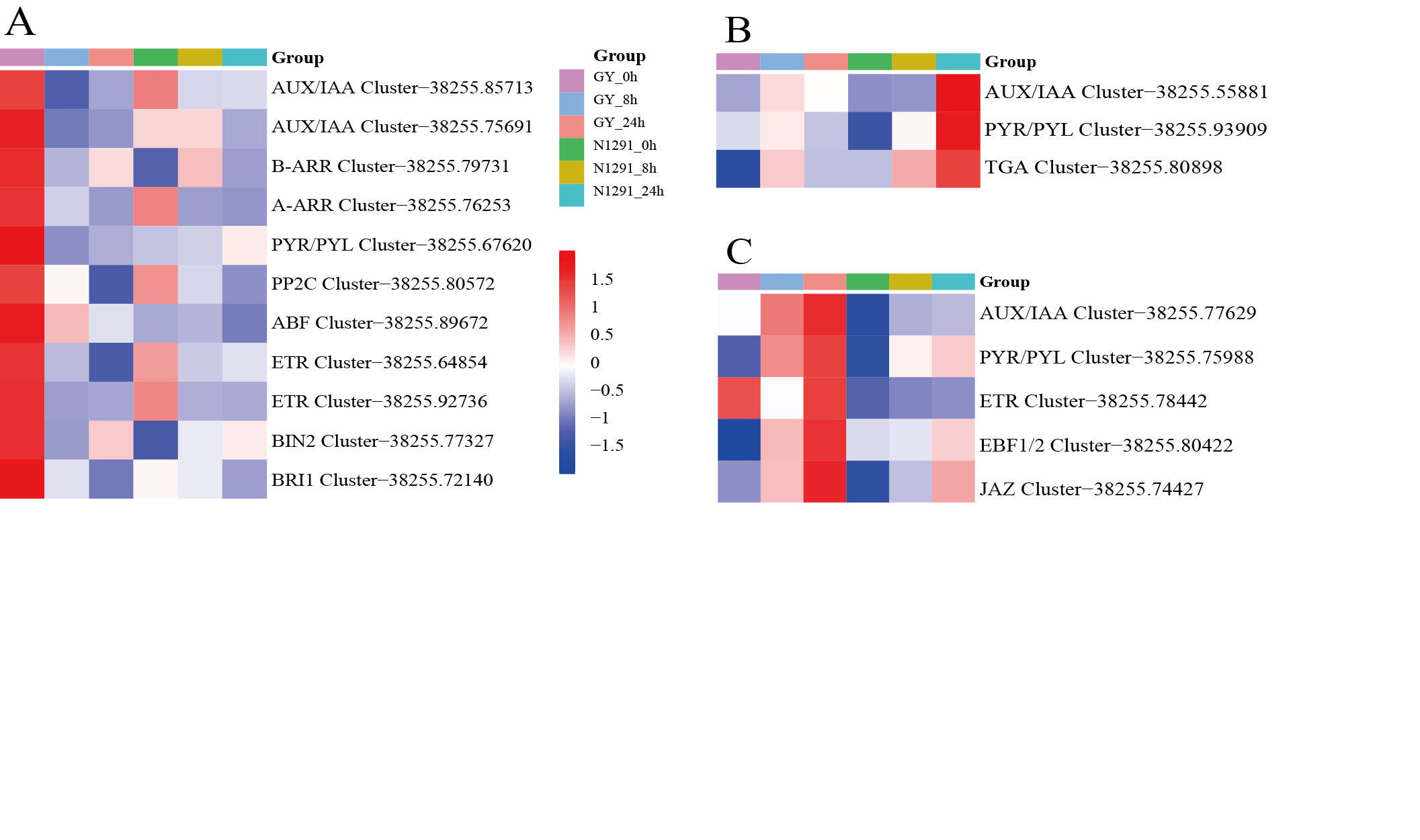


**Supplementary figure 2**. Heatmap analysis of differentially expressed gene clustering in plant hormone signaling pathways. (A-C) blue, green-yellow and purple module respectively.


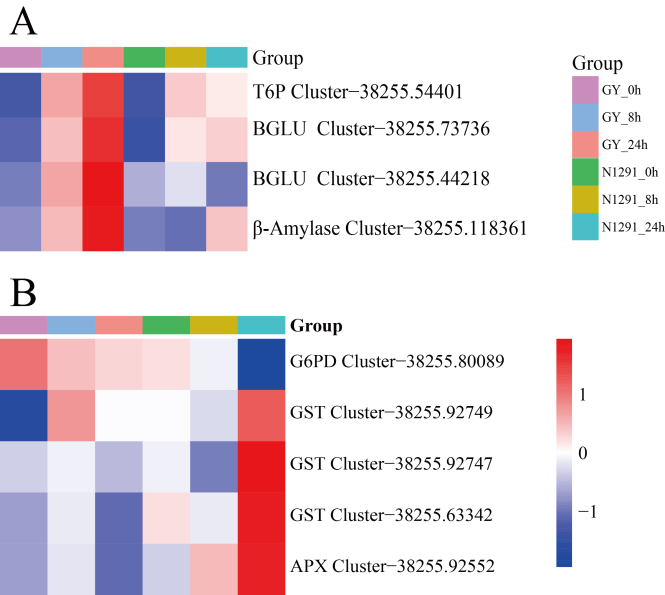


**Supplementary figure 3**. Gene clustering heat map analysis.(A) Clustering analysis of differentially expressed genes of starch and sucrose metabolic pathways (purple module); (B) Glutathione metabolism differentially expressed gene heat map(green-yellow module).
